# A cross-sectional analysis of the effectiveness of a nutritional support programme for people with tuberculosis in Southern Madagascar using secondary data from a non-governmental organisation

**DOI:** 10.1186/s40249-024-01182-8

**Published:** 2024-02-02

**Authors:** Mara Anna Franke, Julius Valentin Emmrich, Fierenantsoa Ranjaharinony, Onja Gabrielle Ravololohanitra, Harizaka Emmanuel Andriamasy, Samuel Knauss, Nadine Muller

**Affiliations:** 1 Global Digital Health Lab at Charité Center for Global Health, Charité – Universitätsmedizin Berlin, Berlin, Germany; 2 London School of Hygiene and Tropical Medicine, London, UK; 3 Ärzte Für Madagaskar E.V., Leipzig, Germany; 4 Heidelberg Institute of Global Health, Heidelberg University, Heidelberg, Germany; 5 Berlin Institute of Health at Charité - Universitätsmedizin Berlin, Berlin, Germany; 6 Doctors for Madagascar, Antananarivo, Madagascar; 7 Speciality Network: Infectious Diseases and Respiratory Medicine, Charité - Universitätsmedizin Berlin, Berlin, Germany

**Keywords:** Tuberculosis, Sub-Saharan Africa, Undernutrition, Nutritional support

## Abstract

**Background:**

There is a strong, bi-directional link between tuberculosis (TB) and undernutrition: TB often causes undernutrition, and undernourished people are more likely to contract TB and experience worse outcomes. Globally, several TB nutritional support programmes exist; however, evidence on their effectiveness is limited and contested. This study evaluates the effect of a nutritional support programme implemented for people with TB in the Atsimo-Andrefana region, Madagascar in 2022. Within this programme, undernourished people with TB [with a body mass index (BMI) of < 18.5 kg/m^2^] receive 0.6 L of vegetable oil and 6.0 kg of a soy-wheat blend per month throughout their TB treatment.

**Methods:**

We analysed secondary non-governmental organisation data collected between January and November 2022 in the Atsimo-Andrefana region, Southern Madagascar, including information on an individual’s medical conditions (e.g., type of TB, treatment outcomes) and nutritional status measured prior to, during, and after completion of treatment (e.g., height, weight, mid-upper arm circumference). We conducted descriptive analyses of patient baseline characteristics and outcomes to assess the impact of the provided nutritional support on the BMI of people with TB.

**Results:**

A total of 1310 people with TB were included in the study [9.9% (130) children under the age of 5, 32.1% (420) children between 5 and 18 years, 58.0% (760) adults]. 55.4% of children under 5, 28.1% of children between ages 5 and 18, and 81.3% of adults were undernourished at treatment initiation. 42.3% (55/130) of children under 5 experienced severe acute malnutrition at treatment uptake. While the average BMI of adults with TB receiving food support increased over time, from 17.1 kg/m^2^ (interquartile range: 15.8–18.3, range: 10.3–22.5) to 17.9 kg/m^2^ (interquartile range: 16.6–19.1, range: 11.9–24.1), most adults remained undernourished even after completing TB treatment.

**Conclusions:**

The current TB nutritional support programme falls short of sufficiently increasing the BMI of people with TB to overcome malnutrition. There is an urgent need to revise the nutritional support available for people with TB, particularly for children under 5.

**Graphical Abstract:**

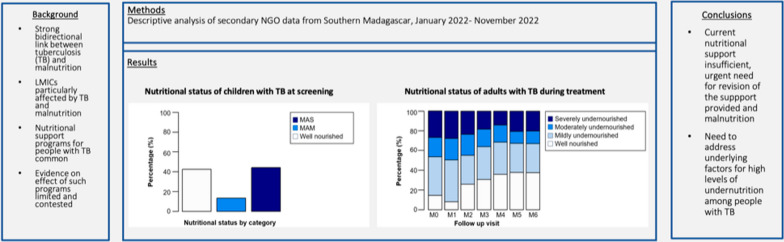

## Background

### Tuberculosis globally

Tuberculosis (TB) remains one of the most prevalent infectious diseases globally: annually, approximately 10.5 million people are affected by the disease, the majority of whom live in low and middle-income countries [1]. Mortality from untreated TB is high, about 50%, and in 2021 alone, 1.6 million people died from the disease globally [1]. The COVID-19 pandemic has significantly set back the fight to end TB, with incidence and prevalence rates rising instead of falling for the first time in years in 2021 [1]. As of 2022, the world falls short of achieving the World Health Organization’s (WHO) goal of a worldwide reduction in TB deaths by 95% and a reduction in TB incidence rate by 90% (i.e., less than 10 TB cases per 100,000 population) by 2035 [2].

### Tuberculosis and malnutrition

There is a strong bidirectional link between TB and undernutrition. On the one hand, people with TB frequently experience concurrent undernutrition. A 2013 systematic review found undernutrition, defined as a body-mass-index (BMI) below 18.5 kg/m^2^, in 20–72% of all people with TB, predominantly in low- and middle-income sub-Saharan African countries (e.g., Tanzania, Malawi, Ghana) [3]. While some people with TB recover from undernutrition during TB treatment, a significant proportion remains undernourished even after completing adequate TB treatment [4–6].

On the other hand, people with undernutrition are at significantly higher risk for both the onset and heightened severity of active TB disease [7–10]. Specifically, undernourished people with TB often experience more extensive disease, such as a greater extent of lung involvement in people with pulmonary TB [11]. They also present longer treatment durations, worse treatment outcomes, and higher relapse rates [12, 13]. Lastly, undernourished people with TB are also at higher risk of TB-associated mortality [12, 14].

Children are especially affected by these co-dependent diseases. Firstly, children are more prone to undernutrition, which makes them particularly vulnerable to its effects [15, 16]. Secondly, diagnosing TB in children is more challenging due to unspecified symptoms, difficulties in obtaining sufficient biological samples for testing, and low bacillary load [17, 18]. TB remains a common complication for undernourished children and the co-occurrence of both diseases predicts significantly worse outcomes. [19]

### Nutritional support programmes for people with TB

WHO recommends universal screening of people with TB for nutritional status and the provision of nutrient-rich or fortified supplementary foods for those with moderate undernutrition. Severe acute undernutrition, especially in children, is recommended to be treated following standard national protocols, as per WHO guidelines. [20]

Consequently, many national TB prevention and care programmes, along with most international agencies active in TB care, have incorporated nutritional support programmes into their TB prevention and care strategies.

The World Food Programme (WFP), a United Nations agency providing food assistance to vulnerable populations, currently offers nutritional support to people with TB in multiple countries, including several in sub-Saharan Africa [21]. The World Bank also funds national TB interventions incorporating nutritional support in various countries, including Papua New Guinea [22], Lesotho, Malawi [23], and India [24]. Equally, national programmes and policies, such as the national TB prevention and care programmes of Mozambique [25] or Kenya [26], have highlighted the need for nutritional interventions for people with TB.

Despite the multitude of nutritional support programmes for people with TB, the evidence on the effectiveness remains limited and contested. Many of these interventions lack scientific evaluations (or the dissemination of their outcomes), resulting in limited scientific evidence on their impact on disease and treatment outcomes. The evidence that does exist is inconclusive. Some studies suggest weight gain among people with TB receiving nutritional support but often lack sufficient sample sizes to show statistical significance [27]. A 2016 systematic review showed that while nutritional support may have a positive effect on weight gain in some settings, it has little to no effect on treatment outcomes [28]. Conversely, a 2015 systematic review of randomised controlled trials reported an increase in successful treatment outcomes and a shorter time to treatment taking effect in people with TB receiving nutritional support [29]. A recent study from rural India reported an adjusted hazard ratio of 0.39 for TB-related mortality among people with TB who received nutritional support and gained at least 5% of their body weight during TB treatment, demonstrating that an increase in body weight is associated with a significantly lower risk of death due to TB [30].

Providing nutritional support to people with TB may offer several other important advantages. Firstly, it can encourage those who avoid seeking care because they fear the indirect economic costs of treatment and their impact on meeting nutritional needs [31–33]. This has been shown to be the case in Madagascar, where nutritional support for people with TB increased detection rates for TB, particularly in rural areas [34]. Secondly, people with TB often face stigma and subsequent social exclusion, leading to potential financial hardships (e.g., because they are excluded from employment opportunities due to stigmatisation) for them and their families [35]. Nutritional support programmes can help mitigate these indirect economic burdens associated with treatment. Thirdly, linking nutritional support provision to healthcare visits might be a way to encourage treatment adherence, an essential factor in avoiding patients being lost to follow up. Lastly, such programmes may have a positive impact on individual well-being [28], a key factor for people with TB, who tend to report lower well-being scores than the general population and frequently suffer from mental health issues. [36]

Considering this conflicting evidence regarding the impact of nutritional support for people with TB, assessing the appropriateness and effectiveness of context-specific policies and interventions is crucial. This not only provides valuable insights for policy design and implementation but also enriches the global knowledge base on nutritional support programmes for patients with TB, ultimately enhancing programme effectiveness.

### Study aims and objective

This study aims to assess the effectiveness of the current WFP nutritional support policy for undernourished people with TB in Southern Madagascar, a region characterised by chronic food insecurity. We thereby aim to provide insights and recommendations to shape the WFP policy for Madagascar and inform nutritional support programmes for people with TB in similar resource-constrained settings.

## Methods

### Study design

This is a retrospective, longitudinal quantitative study, drawing on secondary programmatic data from a TB treatment programme implemented by a non-governmental organisation (NGO) in Southern Madagascar. The data collection spanned from January to November 2022.

### Study setting

Madagascar is an island nation in sub-Saharan Africa, with approximately 29 million inhabitants [37]. It is one of the least developed countries globally, with 81% of its population living below the international national poverty line of 2.15 USD (2017 purchasing power parity) [37]. Over 60% reside in rural areas, with over 74% engaged in the primary sector, predominantly agriculture. [38]

In Madagascar, TB prevalence is high, with 233 cases per 100,000 people (2022 data), notably higher than the global average of 134 cases per 100,000 people [39]. In 2020, only half of the people with TB in Madagascar were notified [1], with effective treatment coverage estimated at only 59% [1]. The reasons for these figures are manifold and include low health literacy [40], long distances to treatment sites, limited diagnostic equipment and drugs, a shortage of trained TB healthcare workers, and TB-associated stigma and social exclusion [41–43]. Lastly, food insecurity poses a significant barrier to accessing and completing TB treatment, as people with TB often experience a loss of livelihood after diagnosis, leading to reduced nutrition intake and creating a cycle of vulnerability. [43]

In Southern Madagascar, encompassing Atsimo-Andrefana, Anosy, and Androy regions, the aforementioned challenges are exacerbated by poverty rates exceeding 90% and specifically weak infrastructure [38, 42]. In 2021 and 2022, the region endured a severe famine, impacting more than 1 million people suffering from acute food insecurity [44]. As of 2023, more than 334,000 people in Southern Madagascar continue to face emergency food insecurity [45]. Concurrently, TB prevalence in the region has been found to significantly surpass the national average, alongside elevated rates of patients lost to follow-up during treatment [41, 46]. In response to these challenges and in accordance with its most recent country strategic plan, the WFP prioritises nutritional support for the most vulnerable, including women, children, and people with TB, specifically in Southern Madagascar [46].

### Intervention

**Fig. 1:**
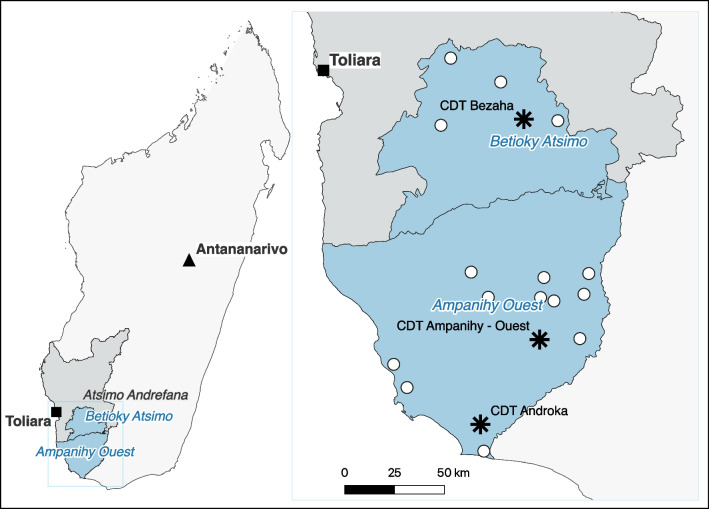
Map of the study zone in the Ampanihy Ouest and Betioky Atsimo districts within the Atsimo-Andrefana region in Madagascar.* Blue markings: intervention districts within the Atsimo-Andrefana region, grey markings: non-intervention districts within the Atsimo-Andrefana region, asterisks: tuberculosis care centers [French centre de diagnostic et de traitement (CDT)], dots: sites where nutritional support was provided, square: capital of the Atsimo-Andrefana region, triangle: capital of Madagascar*

In this setting, the German Malagasy NGO Doctors for Madagascar has been collaborating with Madagascar’s National TB programme since 2019 to promote community-based TB care, aiming to enhance access and improve the quality of TB care for rural populations [41]. The programme is built on four pillars: (1) training and capacity building of healthcare workers across all levels of TB care, (2) facilitation of mobile TB screening and treatment clinics to reach populations in hard-to-reach areas, (3) community outreach through mass sensitisation and collaboration with local leaders to strengthen health literacy and knowledge about service availability, and (4) training and motivation of community health workers (CHWs) for patient screening and follow-up. Since 2020, the programme has partnered with the WFP to provide nutritional support for undernourished people with TB. Eligible people with TB (those with a BMI of less than 18.5 kg/m^2^ at TB screening; designated as timepoint M0) receive monthly rations of 0.6 L of vegetable oil and 6 kg of enriched soy- and wheat-based flour. According to the WFP, these ratios are calculated to include provisions for sharing, for instance, with family members potentially also experiencing malnutrition [47]. The flour rations are calculated to provide up to 939 kcal/day, no precise information is provided by the WFP on the energy content of the vegetable oil. These rations are provided during regular follow-up visits at treatment initiation (M1), after the second (M2), third (M3), fourth (M4), fifth (M5) months, and at treatment completion (M6). Nutritional support is contingent on treatment continuity and attendance at follow-up visits. In 2022 the programme provided nutritional support in the catchment areas of three TB diagnostic and treatment centres in the Atsimo-Andrefana region: Ampanihy-Ouest, Androka, and Bezaha. Figure 1 shows a map of these sites.

### Data collection and cleaning

This study used routine programme data collected on structured data entry forms by the programme team for each patient’s follow-up visit. The data covered basic socio-demographic information (community of residence, travel distance to a screening point, age, gender), clinical details [smear-positive pulmonary TB (TBP +), smear-negative pulmonary TB (TBP−), extrapulmonary TB (TEP)], treatment specifics, laboratory test results, nutritional status measurements [height and weight at the beginning of treatment and at each follow-up visit for adults, weight and mid-upper arm circumference (MUAC) for children], and recorded the type and weight of nutritional support received at each visit, with beneficiaries confirming the reception by signature or fingerprint. Height measurements for children and adults were only taken at screening but not repeated during treatment follow-up or at treatment completion.

A standardised data entry mask (i.e., an interface that asked a standard set of questions to all users to capture patient data) including data checks for completeness and value limits was configured in EpiData [48]. All data were independently transcribed twice by two programme team members, supervised by the project lead. The data, originally in French, were translated to English for analysis. The data were stored in a password-protected database and anonymised before being passed on for analysis.

The research team cross-verified data for discrepancies between entries. Verification was sought from the programme team for any discrepancies and extreme values (e.g., BMI < 12.0 kg/m^2^, BMI variation > 0.5 kg/m^2^ between two consecutive visits). Twenty-seven participants were excluded due to unresolved inconsistencies. Participants with missing data points for weight were included in the analysis, patients with missing data points for socio-demographic data, type of TB, or height were excluded (*n* = 12).

Data were included from all people who were diagnosed with TB between January 1, 2022, and November 30, 2022 in the catchment area of three TB diagnostic and treatment centres (Ampanihy-Ouest, Androka, Bezaha). Patients were included on a rolling basis, meaning that people were at different points of treatment between M0 (the screening visit) and M6 (treatment completion) at the time of data extraction (December 1, 2022). People diagnosed with TB outside the period from January 1, 2022 to November 30, 2022 were excluded.

### Data analysis

Participants were classified into different levels of undernutrition depending on their age.

Adults were classified into well-nourished, undernourished, moderately undernourished, or severely undernourished categories, according to the WFP guidelines [49]: (1) Well-nourished (BMI equal to or above 18.5); (2) Undernourished (BMI below 18.5 and equal to or above 17.0) (hereafter: mildly undernourished); (3) Moderately undernourished (BMI below 17.0 and equal to or above 16.0); (4) Severely undernourished (BMI < 16.0).

Children under 5 were classified based on the National Malagasy guidelines, using both z-score for height-for-weight and mid-upper-arm circumference (MUAC) [50]: (1) Well-nourished with MUAC > 125 mm and a z-score > − 2 standard deviations (SD); (2) Moderate acute malnutrition (MAM) with MUAC between 115 and 125 mm and a z-score between − 3 and − 2 SD; (3) Severe acute malnutrition (SAM) with MUAC < 115 mm and a z-score < − 3 SD.

Children aged 5 to 18 were classified into moderately undernourished, severely undernourished, or well-nourished, according to the National Malagasy reference tables for weight for height [50].

We performed descriptive statistics for participants’ nutritional status and socio-demographic characteristics, including continuous variables that were not normally distributed, medians, and interquartile ranges (IQR). We performed chi-square-tests for nutritional status throughout the course of treatment, for (i) people with TB who received nutritional support through the WFP and those who did not, (ii) male and female people with TB, and (iii) by clinical type of TB (TBP + , TBP−, TEP). All analyses were performed in R Studio (Version: 2023.06.1). [51]

### Ethical approval

Ethical approval was received from the London School of Hygiene and Tropical Medicine ethics committee under registration number 29394. Local approval for the use of secondary data was obtained from the Comité Malgache d’Éthique pour les Sciences et les Technologies (CMEST) in Madagascar on April 26, 2023.

## Results

### Study sample

The final sample included a total of 1310 people with TB, 760 (58.0%) of whom were over 18 years old and 130 (9.9%) were children under 5 years of age, 420 (32.1%) were aged between 5 and 18 years old. Table 1 summarises the participant characteristics.

**Table 1:** Participant characteristics in a study assessing the effectiveness of nutritional support for malnourished people with TB, Southern Madagascar, 2022 (*n* = 1310)

Characteristics	*n* (Proportion)
Gender	
Male	625 (47.7%)
Female	679 (51.8%)
Type of TB	
Pulmonary	692 (52.8%)
Extrapulmonary	520 (39.6%) *447/520 (85.9%) of which among children*
Unknown	98 (7.5%)
Distance (km)	
From residence to treatment site, mean (IQR; range)	7 (1–10; 0–50)
Treatment outcome*	
Completed	603 (46.0%)
Cured	603 (46.0%)
LTFU	22 (1.67%)
Death	3 (0.2%)
Referral	26 (1.9%)
Unknown	53 (4.1%)

^*^Completed Completion of the full 6-month TB treatment course with no smear-microscopy at the end of treatment; *Cure*d Completion of the full 6-month TB treatment course with negative smear-microscopy after treatment completion; *LTFU* Lost to follow-up along the TB treatment course; *Referral* Patient referral to higher level facility, e.g., due to non-response to the standard TB treatment course or other complications

### Nutritional status at treatment initiation

Overall, a significant proportion of the sample was undernourished at screening. The chi-square test showed no significant differences in the likelihood of malnutrition among people with TB depending on gender or clinical type of TB (*P*-values: 0.17 and 0.32 respectively).

### Nutritional status of children

**Fig. 2:**
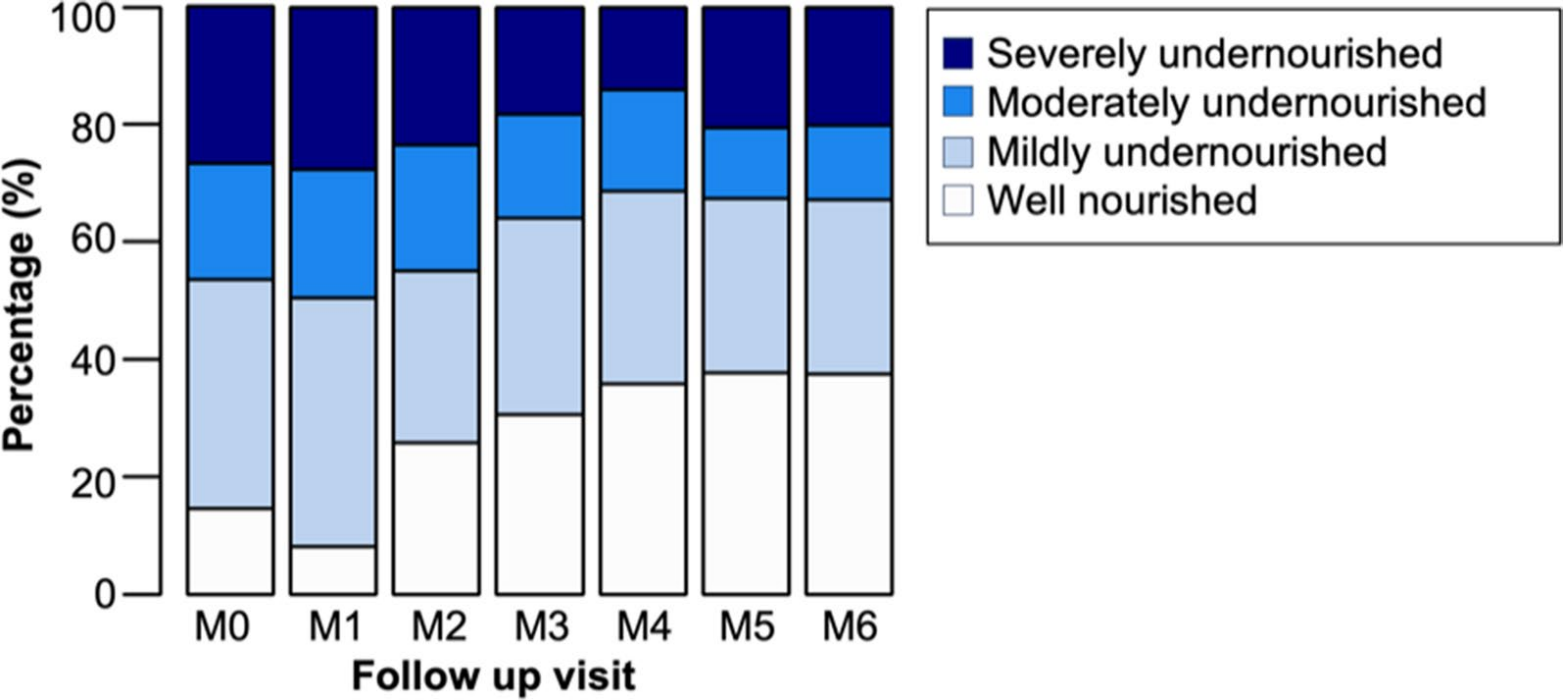
Nutritional status of children younger than 5 years at TB treatment initiation, 2022, Atsimo-Andrefana region, Madagascar. *MAM* moderate acute malnutrition [= Mid-upper-arm circumference (MUAC) 115–125 mm and/or age-specific weight-size indicator between − 3 and − 2 z-scores (Standard deviations (SD) of national reference], *SAM* severe acute malnutrition [= MUAC < 115 mm and/or age-specific weight-size indicator above − 3 and z-scores (SD) of national reference]

Among children under 5 with TB, a total of 55.4% (72/130) were malnourished during the initial TB screening with 42.3% (55/130) exhibiting severe acute malnutrition while 13.1% displayed moderate acute malnutrition (Fig. 2). Between the ages of 5 and 18, 71.9% (302/420) of the sample were well-nourished, 17.1% (72/420) were classified as undernourished, and 10.9% (46/420) as moderately undernourished.

### Nutritional status of adults

In adults, only 18.7% (142/760) were well-nourished at their initial TB screening, while 34.3% (261/760) were mildly undernourished, 17.7% (135/760) were moderately undernourished, and 29.2% (222/760) were severely undernourished. The average BMI at treatment seeking for the entire adult population was 17.1 kg/m^2^ (IQR: 15.8–18.3, range: 10.3–22.5). The average BMI of adults with TB who later received nutritional support (17.0 kg/m^2^) was slightly lower at treatment-seeking than that of those who did not later receive nutritional support (17.1 kg/m^2^).

### Changes in nutritional status over time

Table 2 displays the development of BMI among adults with TB who received nutritional support. There is a noted increase in mean BMI over the 6-month treatment period. However, despite these BMI increases, the overall mean BMI and interquartile ranges remained low, even at treatment completion (M6) (Table 2).

**Table 2:** Changes in body mass index (kg/m^2^) among adults with TB receiving nutritional support during TB treatment in Southern Madagascar, 2022 (*n* = 760)

Timepoint in relation to TB treatment	Body mass index (kg/m^2^)				
	Mean	IQR	Minimum	Maximum	NA (*n*)
M0	17.1	15.8–18.3	10.3	22.5	0
M1	16.9	15.7–18.2	11.9	23.0	0
M2	17.4	16.0–18.6	12.7	23.4	0
M3	17.8	16.4–18.8	12.5	23.5	0
M4	18.0	16.4–19.1	12.5	24.1	0
M5	17.9	16.4–19.2	11.5	23.4	2
M6	17.9	16.6–19.1	11.9	24.1	9

*M0* first screening, *M1* start of treatment, *M2* after first 2 months of treatment, *M3* after 3rd month of treatment, *M4* after 4th month of treatment, *M5* after 5th month of treatment, *M6* end of treatment (after 6th month); *NA* number of participants with missing data

For people with TB not receiving nutritional support, there was also an increase in mean BMI from M0 to M6, with BMI rising from 19.4 kg/m^2^ at M0 to 20.1 kg/m^2^ at M6. However, these increases in mean BMI over time were not statistically significant.

While only 15.1% and 8.3% of the sample were well-nourished at screening (M0) and TB treatment initiation (M1), respectively, this proportion increased to 38.3% by the final treatment visit (M6). It is likely that the average BMI dropped between M0 and M1, as M0 represents the patients’ initial contact with the health system, involving TB screening but not yet receiving treatment, allowing the disease to progress, potentially affecting the patients’ nutritional status. Still, the majority (61.7%) remained undernourished at treatment completion.

**Fig. 3:**
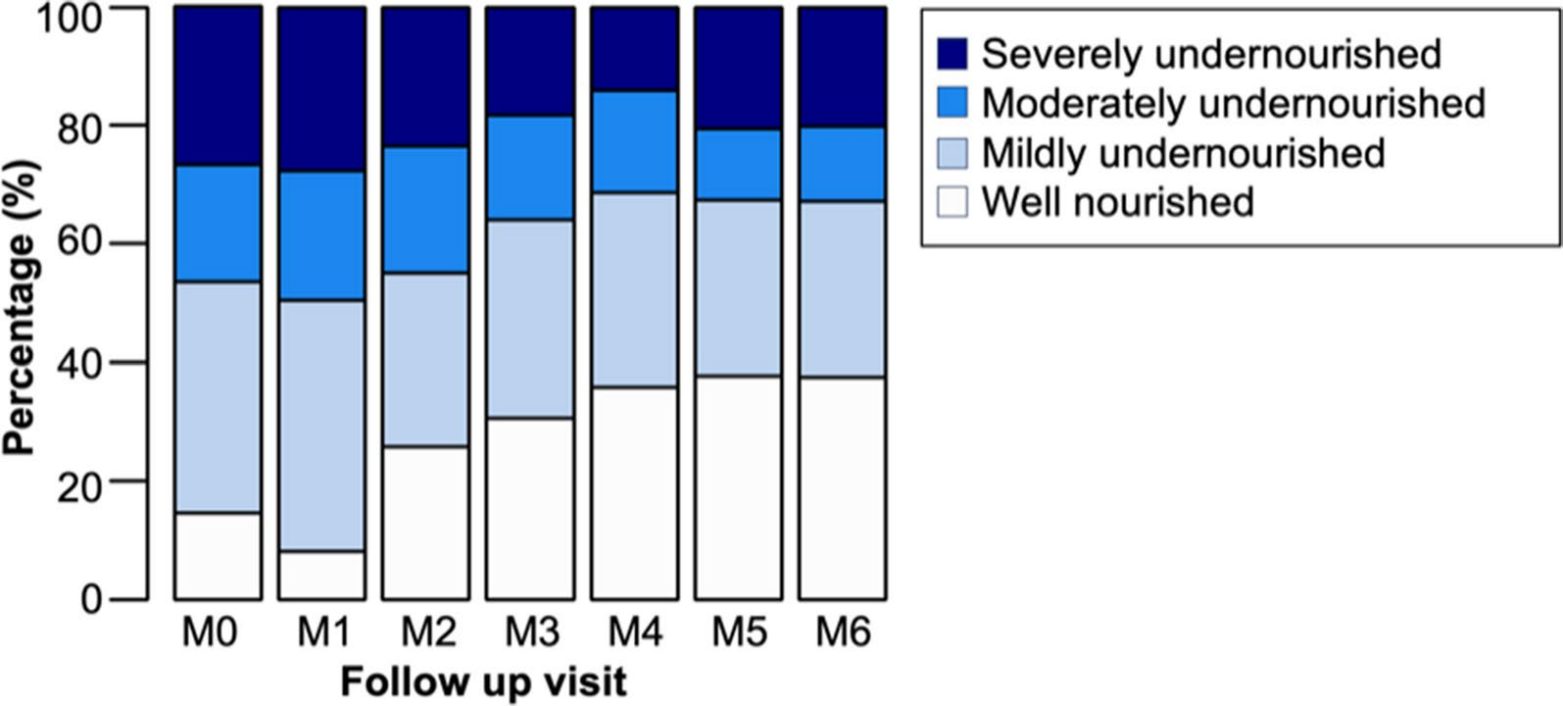
Nutritional status of adults with TB over the course of TB treatment including nutritional support, Southern Madagascar, 2022 (*n* = 760). *Well-nourished* BMI equal to or above 18.5; *mildly undernourished* BMI below 18.5 and equal to or above 17.0; *moderately undernourished* BMI below 17.0 and equal to or above 16.0; *severely undernourished* BMI below 16.0; *M0* first screening, *M1* start of TB treatment, *M2* after first 2 months of TB treatment, *M3* after 3rd month of TB treatment, *M4* after 4th month of TB treatment, *M5* after 5th month of TB treatment, *M6* end of TB therapy (after 6th month)

Further, over 70% of those who had been classified as well-nourished at screening (M0) became undernourished throughout the course of treatment and were classified as undernourished at M6. Figure 3 illustrates the nutritional classification of adults with TB over follow-up visits based on WFP guidelines.

## Discussion

Our findings suggest that the current WFP nutritional support programme does not lead to sufficient weight gain or BMI increases in people with TB in Southern Madagascar to no longer be classified as malnourished. This situation calls for programme adjustments to effectively address the needs of individuals with TB in this chronically food-insecure setting. The standard WFP support package, offering approximately up to 939 kilocalories per day [52], may fall short in meeting the increased caloric demands associated with active TB disease [20]. People with active TB disease have an elevated metabolic rate and, consequently, a higher caloric turnover [20]. Similarly, patients suffering from malnutrition require an increased daily calorie intake to recover from their disease. When both diseases affect a patient simultaneously, the caloric demand is likely to be significantly higher than the average caloric demand of a healthy adult. This inadequacy becomes even more concerning when considering the already alarmingly low nutritional status of most people with TB in this sample, who require additional calories for successful weight gain, even without the burden of TB [53]. To ensure meaningful weight gain for undernourished people with TB in Southern Madagascar, it is imperative to promptly enhance the ration size or energy density of the nutritional support provided.

The undernutrition rate in this programme and setting surpasses that of many other sub-Saharan African countries, where rates typically fall below 50% [3]. Given the dire nutritional situation in Madagascar, particularly in the Atsimo-Andrefana region [45], this finding is both alarming and unsurprising. However, it highlights two paramount imperatives: Firstly, the necessity to address the concurrent diseases of malnutrition and TB in the region, and secondly, the urgent call for comprehensive interventions to enhance overall nutrition in Atsimo-Andrefana. Given the pivotal role of undernutrition and its associated risks for people with TB, combatting widespread undernutrition is indispensable for effectively containing the epidemic in this region.

The high rate of people with TB remaining undernourished after treatment completion (61.7%) aligns with findings from similar studies with high levels of undernutrition at baseline [4, 5]. In these studies, most participants continued to experience undernutrition throughout the treatment period. Notably, these studies were all conducted in regions characterised by high initial undernutrition rates and extreme poverty levels. This might indicate underlying factors that not only affect the likelihood of undernutrition but also of TB and might sustain undernutrition despite successful TB treatment and nutritional support. Low socio-economic status in general and extreme poverty in particular are likely the most relevant underlying factors explaining this observation [3, 10]. Our findings suggest that the current WFP nutritional support programme does not lead to sufficient weight gain or BMI increases in people with TB in Southern Madagascar to no longer be classified as malnourished.

Another alarming finding of this study is the exceptionally high prevalence of severe acute malnutrition (SAM) among children under 5 with TB. With SAM rates exceeding 40% in the study region—well above the 30% threshold that signifies famine according to the Integrated Food Security Phase Classification (IPC) [54]—the situation requires urgent action. SAM already carries a high mortality rate, reaching up to 46% [55], and further increases the risk of mortality rates with TB [56]. While there is a national treatment system for acute malnutrition in children under 5 in Madagascar [50], it appears that this system fails to reach children with TB (in the rural South of the country), resulting in the alarming rates observed in this study. Efforts should focus on strengthening the availability and accessibility of the national treatment system for acute malnutrition for children with TB to ensure timely and adequate care. We furthermore recommend that the nutritional support provided by the WFP for children under 5 with TB should be bolstered to meet their nutritional needs.

Further, the current WFP inclusion criteria only allow people with TB to receive nutritional support if they are classified as malnourished at screening (M0). However, 70% of those adults with TB in our sample who were classified as well-nourished at screening, developed undernutrition throughout the course of treatment. As they do not qualify to receive nutritional support through the WFP under the given inclusion criteria, these people are left without nutritional support and highly vulnerable to the complications caused by malnutrition and TB. To address this challenge and enable access to vital support for this patient group, a revision of the current inclusion criteria appears vital.

Our findings underscore the need for further research to optimise nutritional support for undernourished people with TB. This entails comprehensive, robust studies with solid effect estimates to determine the impact of nutritional support rations on the nutritional status of people with TB. Further investigations into the reasons for the observed lack of weight gain among people with TB are equally important. Factors such as food sharing with family members, the role of stigma, and the influence of concomitant diseases on undernutrition among people with TB, should be elucidated. Analysing the micronutrient status and needs of people with TB, e.g., of iron levels and vitamin D, might be important in tailoring nutritional support programmes for specific regions [57, 58]. Understanding whether micronutrient levels of people with TB differ from those of the general population might be equally important, considering micronutrients’ impact on disease susceptibility [58]. Potentially, this could even indicate important strategies for disease prevention in the long term.

We acknowledge the limitations in our study. The utilisation of secondary programmatic data, not initially collected for research purposes, may have impacted data quality. To mitigate this, we implemented measures such as double data entry including checks and controls, along with manual verification of inconsistencies and extreme data points. In addition, children's height was measured only once at the start of the treatment, which, considering their potential rapid growth, may introduce bias and potentially underestimate undernutrition levels at the end of treatment. The absence of follow-up height measurements in children prevented us from assessing their nutritional status throughout and at the end of treatment, thereby limiting the understanding of the impact of nutritional support on them. Furthermore, the use of non-height-dependent indicators like weight-for-age, which captures both acute (“wasting”) and chronic undernutrition (“stunting”), may have introduced bias, especially in Madagascar with its high prevalence of stunted children. [15, 59]

## Conclusions

Our study shows that the current nutritional support offered for people with TB in the Atsimo-Andrefana region is largely insufficient, emphasising the need for more extensive interventions to effectively address malnutrition in this population. To our knowledge, this study is the first to analyse the nutritional support provided by the World Food Programme in the region, underscoring the necessity to allocate additional resources and attention to the nutritional needs of people with TB, especially children under 5.

## Data Availability

The datasets used and/or analysed during the current study are available from the corresponding author upon reasonable request.

## Abbreviations

BMI: Body mass index
CHW: Community health worker
LTFU: Lost to follow-up
MAM: Moderate acute malnutrition
MUAC: Mid-upper arm circumference
NGO: Non-governmental organisation
SAM: Severe acute malnutrition
TB: Tuberculosis
TBP: Pulmonary tuberculosis
TEP: Extrapulmonary tuberculosis
UN: United Nations
USD: United States Dollars
WHO: World Health Organization
WFP: World Food Programme
